# A triple-classification for the evaluation of lung nodules manifesting as pure ground-glass sign: a CT-based radiomic analysis

**DOI:** 10.1186/s12880-022-00862-x

**Published:** 2022-07-27

**Authors:** Ziyang Yu, Chenxi Xu, Ying Zhang, Fengying Ji

**Affiliations:** 1 Department of Radiology, The First Affiliated Hospital of Harbin Medical University, Harbin, Heilongjiang Province People’s Republic of China; 2 School of Medicine, Xiamen University, Xiamen, Fujian Province China

**Keywords:** Lung adenocarcinoma, Pulmonary nodules, Radiomics, Random forest classification, Computed tomography

## Abstract

**Objectives:**

To construct a noninvasive radiomics model for evaluating the pathological degree and an individualized treatment strategy for patients with the manifestation of ground glass nodules (GGNs) on CT images.

**Methods:**

The retrospective primary cohort investigation included patients with GGNs on CT images who underwent resection between June 2015 and June 2020. The intratumoral regions of interest were segmented semiautomatically, and radiomics features were extracted from the intratumoral and peritumoral regions. After feature selection by ANOVA, Max-Relevance and Min-Redundancy (mRMR) and Least Absolute Shrinkage and Selection Operator (Lasso) regression, a random forest (RF) model was generated. Receiver operating characteristic (ROC) analysis was calculated to evaluate each classification. Shapley additive explanations (SHAP) was applied to interpret the radiomics features.

**Results:**

In this study, 241 patients including atypical adenomatous hyperplasia (AAH) or adenocarcinoma in situ (AIS) (n = 72), minimally invasive adenocarcinoma (MIA) (n = 83) and invasive adenocarcinoma (IAC) (n = 86) were selected for radiomics analysis. Three intratumoral radiomics features and one peritumoral feature were finally identified by the triple RF classifier with an average area under the curve (AUC) of 0.960 (0.963 for AAH/AIS, 0.940 for MIA, 0.978 for IAC) in the training set and 0.944 (0.955 for AAH/AIS, 0.952 for MIA, 0.926 for IAC) in the testing set for evaluation of the GGNs.

**Conclusion:**

The triple classification based on intra- and peritumoral radiomics features derived from the noncontrast CT images had satisfactory performance and may be used as a noninvasive tool for preoperative evaluation of the pure ground-glass nodules and developing of individualized treatment strategies.

**Supplementary Information:**

The online version contains supplementary material available at 10.1186/s12880-022-00862-x.

## Introduction

Lung adenocarcinoma (AD), as the most common histological type of peripheral lung cancer with manifestation of imageology-ground glass nodules (GGNs) on CT images [1, 2], is divided into preinvasive adenocarcinoma (including atypical adenomatous hyperplasia (AAH) and adenocarcinoma in situ (AIS)), minimally invasive adenocarcinoma (MIA) and invasive adenocarcinoma (IAC) according to the 2015 World Health Organization (WHO) classification [3]. Different pathological grades of AD have different degrees of malignancy and disease-free survival. Several studies have validated that the 5-year survival rate for preinvasive AD and MIA has reached nearly 100%, while that for IAC ranges from 38 to 74.6% [4, 5]. Some investigators suggested that the preinvasive status could be followed up conservatively and that MIA could receive limited surgical resection instead of lobectomy [6, 7]. To avoid unnecessary surgery or biopsies, the preoperative classification of AD is of significance in determining the treatment strategy. One significant clinical problem is the noninvasive evaluation of GGNs, which includes the differential diagnosis of benign and malignant nodules and assessment of the pathological degree of malignancy.

To date, nonenhanced chest computerized tomography (CT) is the preferred and most common noninvasive preoperative method to screen lung diseases. With the improvement in the resolution and slice thickness of computed tomography scanners, GGNs have been gradually detected in lung CT images [8]. In current CT-based practice, the main reliance is on the visual evaluation of the shape, size and surrounding conditions of GGNs by radiologists [9, 10]. Due to subjective factors, the diagnostic accuracy showed poor performance. A noninvasive method for the evaluation of GGNs preoperatively is essential to guide clinical management [11].

Radiomics is a technology that characterizes the GGNs by gathering mineable high-throughput features, followed by the machine learning method to select the features related to the final diagnostic model [12, 13]. Radiomics can quantitatively analyse the inherent heterogeneity of GGNs and has been broadly used in the evaluation of pulmonary nodules [14]. Additionally, radiomics features quantifying the peritumoral tissues were related to the degree of invasion [15]. Recent radiomics studies have focused on differentiating the benign and malignant or invasive characteristics of GGNs by traditional dichotomies [16, 17]. In contrast to previous studies, we built a triple-classification radiomics model for the differentiation of precancerosis, MIA and IAC with the manifestation of GGNs based on the combination of radiomics features extracted from intra- and peritumoral tissues.

In addition, previous studies lacked the interpretability of radiomics models, which led to skepticism about the biological mechanism. In our investigation, we explained our classifiers by the Shapley additive explanations (SHAP) framework to increase the usability [18]. SHAP is currently the most recommended for model explanation. First, a weight value is assigned to each feature in the model. These values are then calculated for each prediction independently, and high absolute SHAP values indicate importance, whereas values close to zero indicate low. To our knowledge, this is the first study to build a triple classification with an interpretable radiomics model for the evaluation of GGNs.

The aim of this study was to develop and validate an interpretable triple classification radiomics classifier that may be used as a noninvasive tool for the individual preoperative evaluation of pure ground-glass nodules.

## Materials and methods

### Patients

**Fig. 1:**
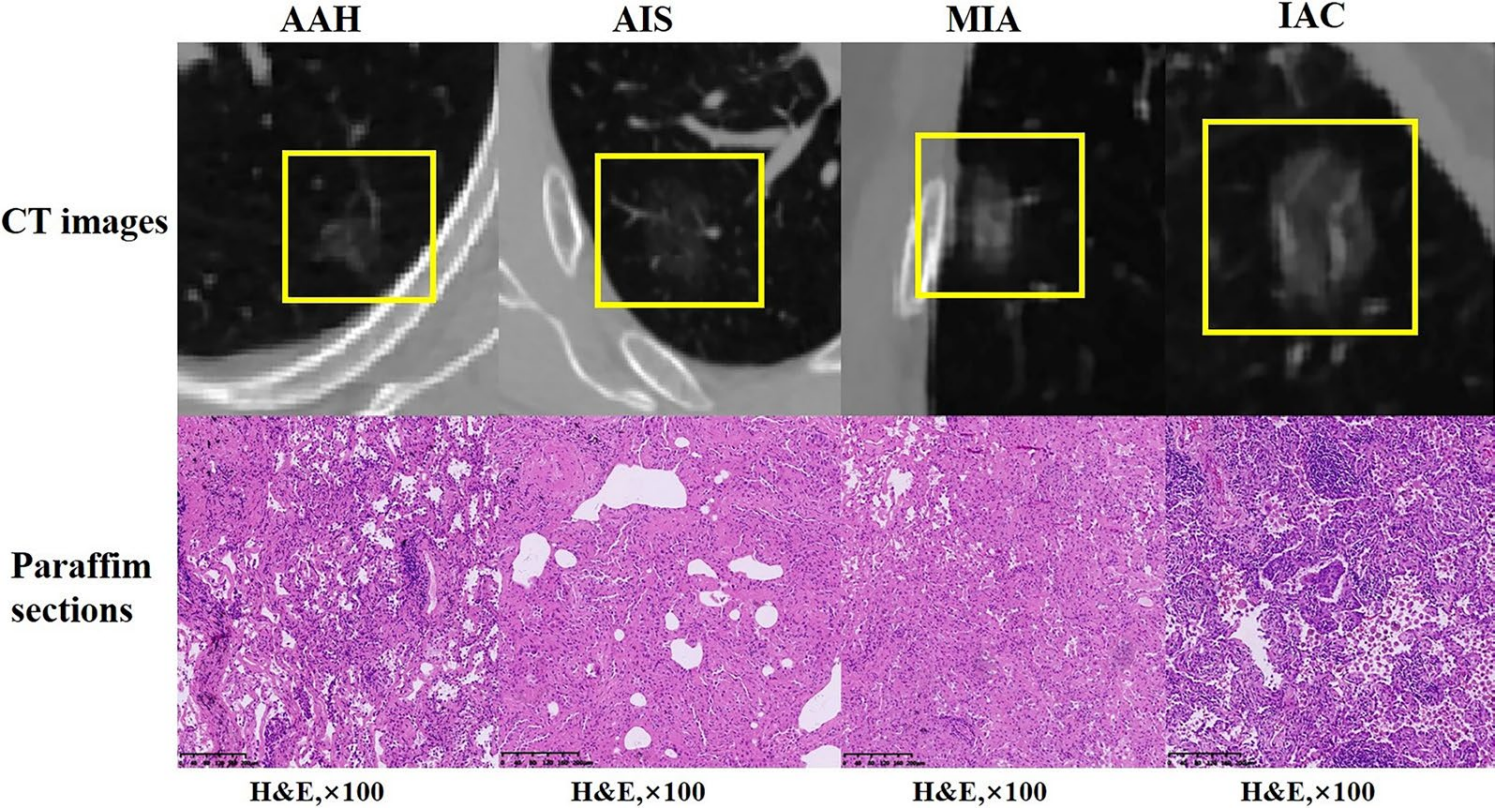
Examples in the dataset of GGNs. The CT images and paraffin sections from left to right (haematoxylin and eosin, H&E, ×100) are AAH, AIS, MIA, and IAC, respectively

**Fig. 2:**
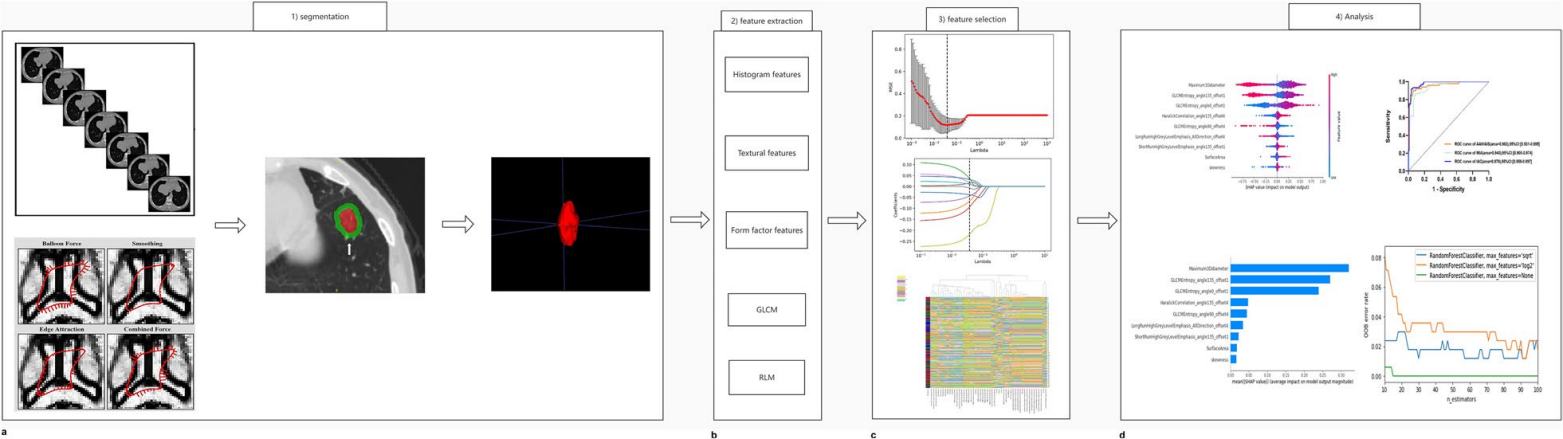
Workflow of radiomics

This retrospective study was approved by the institutional review board of Harbin Medical University, and the requirement for patient informed consent was waived. We retrospectively reviewed the medical charts and CT images between June 2015 and June 2020 from the Picture Archiving and Communication System (PACS). The inclusion criteria in the analysis were as follows: (1) the GGNs were confirmed by postoperative pathology (Fig. 1); (2) the computed tomography findings were pure ground glass density nodules with no solid component; (3) chest CT scans were performed within one week before biopsy or surgery; and (4) the CT images included in the study were all taken from the same CT device (GE Discovery CT750 64-detector CT scanner). The exclusion criteria were as follows: (1) subsolid nodules; (2) obvious calcifications in nodules; (3) images that had significant noise or artefact; (4) lesions less than 1.0 cm, where the region of interest (ROI) could not be accurately delineated; and (5) patients who had a biopsy before the CT. The training cohort was the patients between June 2015 and June 2019, and the independent testing cohort included patients between July 2019 and June 2020.

### CT image acquisition

All patients were scanned using a GE Discovery CT750 64-detector CT scanner (GE Medical Health care, Milwaukee, Wisconsin) with a tube voltage of 120 kV and a tube current of 80 mA with auto exposure control; pitch 0.875–1.5; detector collimation 0.625–2.5 mm; and field of view (FOV) 360 mm × 360 mm. The scan included the entire thorax with a thickness of 1.0 mm per layer. Single scans were obtained during deep inspiration and breath hold. Lung images were reconstructed with the use of a high-spatial frequency algorithm, and mediastinal images with the use of an intermediate-spatial-frequency algorithm.

### Region of interest segmentation

CT images were evaluated at the appropriate diagnostic lung window (level, − 450 HU; width, 1500 HU). As Fig. 2a shows, the intratumoral volume of interests (VOI) was semiautomatically segmented on serial axial CT images by the software package ITK-SNAP version 4.11.0 (www.itk-snap.org) in two steps. First, label points are marked by one radiologist (Ying Zhang, with 8 years of experience in lung diagnosis). Thereafter, all VOIs are generated automatically by computing devices, based on the label points. After segmentation, the peritumoral VOIs were created at a distance of 15 mm outside of the lesions according to the morphology by two radiologists [19]. The results were identified by one experienced radiologist (Ji, with over twenty years of experience in lung diagnosis).

### Radiomics feature extraction

We performed the feature extraction for each discretization using the AK software (Artificial Intelligence Kit; version V3.2.0; GE Healthcare, China, Shanghai). Each ROI provided 282 texture features derived from the two types of features. One is the absolute signal quantisation including histograms (first order statistics, 42 features) and form factors (three-dimensional size and shape, 9 features). The other is the relative signal quantisation, which contained texture parameters (appearance of the surface, 40 features), grey-level cooccurrence matrix (GLCM, 71 features) and run-length matrix (RLM, 120 features) obtained using four angles (0°, 45°, 90° &135°) and two displacement vectors (1 & 4 pixel) (Fig. 2b). A total of 203 886 texture feature values for each discretization was calculated. Texture features and the number of discretisation levels are listed in Additional file 1: Table S1. The radiomics features were consistent with the Imaging Biomarker Standardization Initiative (IBSI). The mathematical definitions are based on the previous studies [20, 21]. First, normalization (z score transformation) was performed on the imaging data to avoid dimension bias, and then we used absolute values to further compare weights. The reproducibility of the extracted features was measured by intraclass correlation coefficients (ICCs). We randomly selected 30 patients, and the interobserver reproducibility was assessed by two radiologists (Ying Zhang and ChenXi Xu). Subsequently, the radiologist (Ying Zhang) reperformed the VOI on these 30 patients after five days. Only the features with ICC > 0.80 were considered to be retained for subsequent analysis.

### Feature selection

The process of radiomics analysis is shown in Fig. 2c. To avoid redundant data, one-way ANOVA with a familywise error (FWE) correction was applied to select features in the training set. Features were considered important at FWE-cor. *p* < 0.05. Subsequently, the selected radiomics features were ranked using the minimum redundancy maximum relevance (mRMR) algorithm, which selects features by minimizing the redundancy and maximizing the correlation between the features. In our study, the first 25% features calculated by mRMR were reserved [22, 23]. Next, the selected radiomics features were analysed by least absolute shrinkage and selection operator (LASSO) regression, a method for feature selection in super dimensional data. The parameter λ penalty of the regression was determined by using a grid search on tenfold cross-validation according to the minimum mean squared error (MSE) in the training set.

### Classification and evaluation

The random forest (RF) algorithm was used in our study for triple classification (Fig. 2d). To avoid model overfitting, the model was constructed using tenfold cross-validation in the training cohort. The process of RF is to generate multiple independent “classification and regression trees” based on the selected features with automated and randomized decision points. Then, the bootstrap method was used to randomly divide the sample sets to fill the decision points. In addition, the ‘out-of-bag’ (OOB) data, as the samples that were not included in the “bootstrap sample”, were subsequently used to internally validate the accuracy of the derived RF classifier. The features were randomly selected as candidates for each cut-off in the decision trees and were selected by calculating the Gini index [24]. Based on this calculation method, a set of candidate features with excellent reproducibility and significant differences were generated for the final multiple decision trees. The two key parameters were set according to their stability and best performance by the ‘Grid Search CV’ algorithm (60 estimators, 7 max features, minimum 7 samples on a leaf node). The processes above were performed in the Anaconda3 platform (www.anaconda.com) with the “scikit-learn” package (scikit-learn.org). The parameter class-weight was set as ‘balanced' to avoid sample bias.

The performance of the classifier was evaluated on the testing set which were independent of the training set without any preprocessing. We also evaluated and compared the potential of CT-based radiomics in identifying three groups, AAH/AIS, MIA and IAC. Receiver operating characteristic (ROC) curve analysis and the area under the ROC curve (AUC) with 95% CI, sensitivity, specificity values were calculated to evaluate the effectiveness of the models on the validated and test sets.

To improve the interpretability of the machine learning models, we calculate the SHAP value of each feature to explain the prediction for the model. For each predicted ROI, the contribution of each feature to the RF model was allocated based on their contribution, and the SHAP values were generated based on the axioms.

## Statistical analysis

Analysis of variance (ANOVA) was used for the radiomics features of the three groups, and post hoc testing was applied for the analysis of pairwise differences. Statistical analyses were performed using SPSS (version 25, Chicago, IL, USA). A two-tailed p value less than 0.001 was considered statistically significant. The statistical significance of the balanced accuracy was computed by the permutation test (iteration 1,000 times) in Python version 3.7.4.

## Results

### Patient characteristics

At a ratio of 7:3, 168 patients were included in the training cohort (50 AAH/AIS, 58 MIA, and 60 IAC). In the testing cohort, 73 patients (22 AAH/AIS, 25 MIA, and 26 IAC) were enrolled based on the stratified sampling method. There were no significant differences in age or sex in the three groups in either the training or testing cohorts. The clinical characteristics are presented in Table 1.

**Table 1:** Demographic characteristics of AAH/AIS, MIA and IAC patients in the training and testing cohorts

Characteristics (mean ± SD)	Training set (n = 168)			*p* value	Testing set (n = 73)			*p* value
	AAH/AIS	MIA	IAC		AAH/AIS	MIA	IAC	
Age (years)	44.66 ± 7.80	44.36 ± 7.27	44.17 ± 7.34	0.638	45.41 ± 7.68	44.88 ± 6.84	45.08 ± 7.32	0.881
Gender (male/female)	23/27	25/33	31/29	0.700	10/12	13/12	12/14	0.701

*SD* standard deviation

### Performance of radiomics feature selection and model construction

A total of 846 radiomics features were calculated for three VOIs, each including 42 histogram, 9 form factor, 40 texture parameters, 71 grey level cooccurrence matrix (GLCM), and 120 run-length matrix (RLM) features. A total of 219 ineligible features with ICCs less than 0.8 were excluded. Subsequently, 161 features were selected after performing one-way ANOVA with FWE, and 8 valuable features were finally determined based on the mRMR and 10 cross-validation Lasso regression (λ = 3.76E−02). The representative radiomics features are shown in Table 2. The RF classifier was built based on these features for triple-class classification.

**Table 2:** The representative radiomics features

Radiomics features	AAH/AIS	MIA	IAC	F-value/P-value
Maximum3Ddiameter	1.82E+01 ± 1.66E+00	3.72E+01 ± 3.55E+00	5.65E+01 ± 4.07E+00	1.81E+03/***
GLCMEntropy_angle0_offset1	3.72E+00 ± 2.74E+00	5.24E+00 ± 1.30E+00	1.27E+01 ± 1.76E+01	3.33E+02/***
GLCMEntropy_angle135_offset1	4.90E+00 ± 6.82E−01	9.02E+00 ± 8.56E−01	1.43E+01 ± 5.75E−01	2.43E+03/***
HaralickCorrelation_angle90_offset7	8.60E+08 ± 2.04E+08	6.35E+08 ± 2.09E+08	5.29E+08 ± 1.33E+08	4.54E+01/***

***p value less than 0.001

**Fig. 3:**
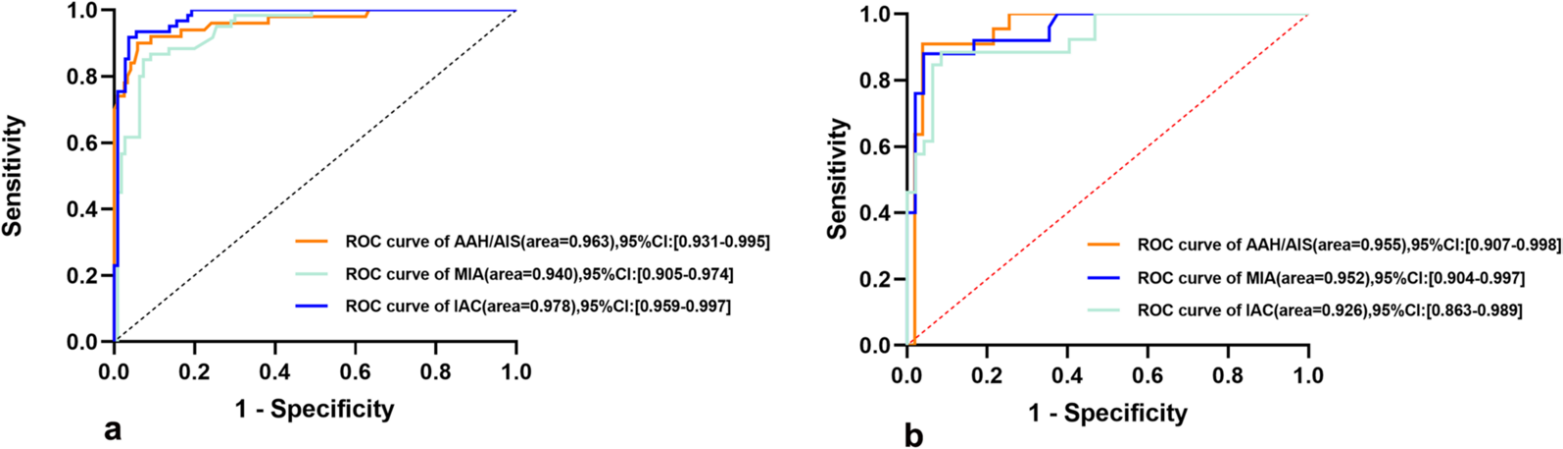
Receiver operating characteristic (ROC) curves of three radiomics models in both the training (**a**) and testing cohorts (**b**)

**Fig. 4:**
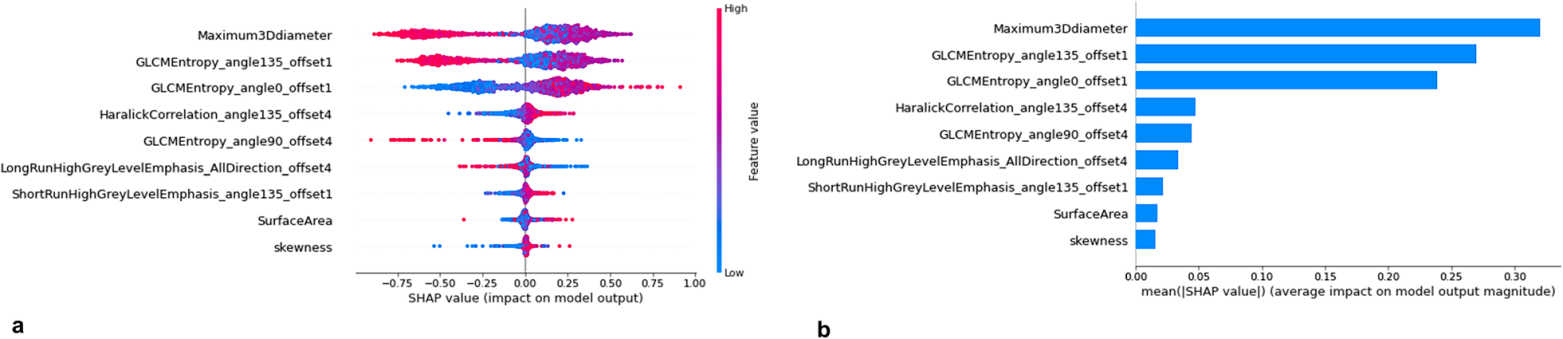
Summary plot of features impact on the prediction of the SVM model. The Shapley additive explanations (SHAP) values of features in every sample

The mean AUCs of the triple-class RF models for AAH/AIS, MIA and IAC yielded values of 0.963 (95% CI 0. 931–0.995), 0.940 (95% CI 0.905–0.974), and 0.978 (95% CI 0.959–0.997), in the training set and 0.955 (95% CI 0.907, 0.998), 0.952 95% CI 0.904, 0.997), and 0.926 (95% CI 0.863, 0.989) in the testing set. The ROC curves of the model in the training and testing sets are shown in Fig. 3a, b and Table 3. To evaluate the feature importance for the classification model, the SHAP values of the selected feature for each prediction were computed in the training set. A positive SHAP value indicated a high likelihood of a diagnosis of the higher pathological grade of GGNs (Fig. 4a, b).

**Table 3:** The diagnostic performance of the radiomic models in both the training and testing sets

Classifier evaluation	Training set (n = 168)			Testing set (n = 73)		
	AAH/AIS	MIA	IAC	AAH/AIS	MIA	IAC
Average AUC	0.963	0.940	0.978	0.955	0.952	0.926
(95% CI)	(0.931,0.995)	(0.905,0.974)	(0.959,0.997)	(0.907,0.998)	(0.904,0.997)	(0.863,0.989)
Average balanced accuracy (%)	0.921	0.893	0.941	0.935	0.919	0.903
Average sensitivity (%)	0.900	0.850	0.918	0.909	0.880	0.885
Average specificity (%)	0.942	0.936	0.963	0.961	0.958	0.915

*AUC* area under the curve

### Analysis of representative radiomics features

After assembling the one-way ANOVA, mRMR, Lasso regression, RF with SHAP and post hoc-ANOVA, the representative radiomics features were finally identified, which included one histogram (intratumoral feature), one Haralick parameter (intratumoral features) and two GLCM parameters (including one intratumoral feature GLCMEntropy_angle0_offset1 and one peritumoral feature GLCMEntropy_angle135_offset1).

**Fig. 5:**
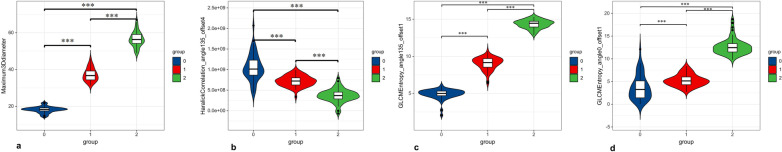
The distributions of representative radiomics features and the post-hoc statistics results in the three groups. *** denotes statistical significance, *p* < 0.001. Class 0 represents AAH/AIS; Class 1 represents MIA; and Class 2 represents IAC

Figure 5 shows the results of the representative radiomics features. The histogram of the Maximum3Ddiameter is measured as the largest pairwise Euclidean distance between the voxels on the surface of the tumour volume. The values (1.82E+01 ± 1.66E+00 in AAH/AIS; 3.72E+01 ± 3.55E+00 in MIA, 5.65E+01 ± 4.07E+00 in IAC, *p* < 0.001) in IAC patients were the highest and lowest in AAH/AIS (Fig. 5a). The texture featureHaralickCorrelation_angle135_offset4 measures the degree of similarity of the grey level of the image in the row or column direction. When compared with that in MIA and IAC patients, the value shown in Fig. 5b (1.05E+09 ± 3.24E+08 in AAH/AIS; 7.20E+08 ± 1.31E+08 in MIA, 3.68E+08 ± 1.62E+08 in IAC, *p* < 0.001) were highest in AAH/AIS patients.

The GLCM feature represents the joint probability of certain sets of pixels having certain grey-level values, and entropy measures the loss of information or the message in a transmitted signal as well as the image information. The intratumoral GLCMEntropy_angle0_offset1 (3.72E+00 ± 2.74E+00 in AAH/AIS, 5.24E+00 ± 1.30E+00 in MIA, 1.27E+01 ± 1.76E+01 in IAC, *p* < 0.001) and the peritumoral GLCMEntropy_angle135_offset1 (4.90E+00 ± 6.82E−01 in AAH/AIS, 9.02E+00 ± 8.56E−01 in MIA, 1.43E+01 ± 5.75E−01 in IAC, *p* < 0.001) were both higher in IAC patients than in benign groups (Fig. 5c, d).

## Discussion

The evaluation of GGNs, which are defined as slightly dense nodules without blocking other tissues on CT images, has been a challenge for clinicians. Many benign pulmonary diseases, such as inflammation, AAH or AIS, are often mistaken for lung cancer and undergo unnecessary surgery due to overlapping imaging characteristics [25]. In addition, for malignant GGNs, preoperative evaluation of invasion is significant for individual treatment. In our study, we developed and validated a machine learning model based on intratumoral and peritumoral radiomics features for the noninvasive assessment of GGNs on CT images, which exhibited good performance. The present study is the first to build a visual triple classifier by the SHAP algorithm using radiomics features derived from CT images to identify the status of GGNs. With the representative radiomics factors, the classifier demonstrated impressive efficiency with an average AUC of 0.935 in the training set, which is important for accurately assessing GGNs.

Previous studies have analysed CT-based radiomics features andmorphology in assessing GGNs. Fan et al. identified the importance of texture in the evaluation of the invasive degree of GGNs [17]. They found that the radiomics feature model had good performance in predicting the extent of GGNs invasion (AUC value of 0.936). However, their study lacked indolent GGNs in situ, which could be observed during follow-up. Based on radiomics, Sun et al. combined traditional morphological features, such as size, to establish a model to predict invasive lesions [24]. Although the combined model improved diagnostic accuracy, it also increased the workload and was prone to subjective error. In addition, Chen et al. developed a radiomics nomogram to differentiate lung adenocarcinomas and benign granulomatous lesions; however, early-stage cancers were not included in their study, which has become the focus of clinical attention [16]. For GGNs, previous investigations have focused on evaluating of the differentiation between benign and malignant lesions or the degree of invasion [26]. However, clinicians are more concerned with the specific biological characteristics of GGNs that determine the subsequent therapeutic strategies. Although Meng et al. preoperatively evaluated the invasiveness of pulmonary adenocarcinomas manifesting as GGNs, they compared only two groups [27]. Our study, for the first time, built a triple classification based on intra- and peritumoural CT radiomics features to comprehensively predict GGNs. According to the SHAP values, the most representative CT radiomics features that were correlated with the pathological grade of GGNs were analysed among the three groups. After one-way analysis of variance, the significant features consisted of one histogram, one textural parameter and two GLCM parameters. The histogram feature described the basic characteristics of the VOIs. Our study found that the histogram-Maximu3Ddiameter was highest in the ICA group and the lowest in the preinvasive group, which was associated with malignant behaviour. Sun et al. also confirmed that the size of traditional CT morphology was significantly different between invasive and noninvasive groups [24]. Wu et al. found that the size of the lesion has limited performance between benign and malignant lung lesions [28]. The measurement of tumour size in previous studies was mainly based on the maximum diameter of the image axis, which may lead to subjective bias. In our study, the parameter-Maximum3Ddiameter could measure the largest pairwise Euclidean distance between voxels on the surface of the tumour volume and was more consistent with the actual characteristics. We also found that the entropy of the GLCM is related to the pathology grade of GGNs. The entropy of the GLCM is the texture feature that represents the randomness of intensity and spatial heterogeneity. For pathological grade, AAH or AIS are localized to inert lesions with noninvasive biological behaviour. In contrast, IAC consists of multiple compartments that may result in a large range of intensity. A growing number of studies have proven that GLCM features may play an important role in reflecting pathological invasion and the composition of lesions [29, 30]. In our study, the values of GLCM entropy were in descending order among IAC, MIA and AAH/AIS, which reflected that the texture features of the images are disordered with the deterioration of biological behaviour. Furthermore, the Haralick parameter was also analysed in our context. Recently, some scholars have confirmed that the Haralick parameter is a stable and reliable index in texture analysis [31, 32]. The Haralick parameter correlation is used to measure the direction of the greyscale and represent the correlation of grey values among neighbouring voxels. The parameter was found to be lowest in IAC patients and highest in AAH/AIS patients suggesting heterogeneous subcompartmental decomposition and microscopic infiltration in the IAC group. In our study, the radiomics features served as objective indicators to evaluate the composition of the GGNs and predict the degree of pathological invasion preoperatively.

Furthermore, we built a random forest classifier on the basis of these contributing features. The random forest method was first invented by Ho in 1995 and was proven in recent years to be very efficient and effective in sorting through high-dimensional data and especially suitable for triple classification. In recent years, RF has been applied to various body systems in medical images and is suitable for screening texture parameters [33, 34]. Several studies have focused on the application of machine learning-aided approaches for the diagnosis of lung tumours. Cho et al. built three classifications to differentiate IAC from MIA. The best performance was achieved by the logistic model, an algorithm that might be suited for predicting the risk of a single event [35]. For multiple classifications, Wang et al. built an RF model for predicting peripheral lung cancer presenting as GGNs [36]. In contrast to the previous study, the parameters of the RF model in our study were selected by the “Grid Search” CV method according to the best performance of the total out-of-bag error based on the 10 cross-validation, and a permutation test was used to confirm the learning outcomes. Hence, the RF model based on radiomics features in our study had average AUCs of 0.960 and 0.944 in the training and testing sets, respectively.

### Generalizability issues and limitations

There were some limitations to the present study. First, although our study included 241 patients, the sample size was relatively small. Second, the study design was only one centre and lacked an independent dataset for cross-validation. Therefore, multicentre with larger case numbers are required to further validate in our future work. In addition, future investigations might combine radiomics with genomics.

## Conclusions

In conclusion, we proposed a triple random forest model to facilitate the preoperative evaluation of GGNs. The triple classification based on intra- and peritumoral radiomics features derived from noncontrast CT images had a satisfactory performance, which may be used as a noninvasive tool for the individual preoperative evaluation of pure ground-glass nodules.

## Supplementary Information

**Additional file 1**. The detail of feature selection.

## Data Availability

The data used to support the findings of this study are available from the corresponding author upon reasonable request. The data that support the findings of this study are available from the First Affiliated Hospital of Harbin Medical University but restrictions apply to the availability of these data, which were used under license for the current study, and so are not publicly available. Data are however available from the corresponding author upon reasonable request and with permission of the First Affiliated Hospital of Harbin Medical University.

## Abbreviations

AAH: Atypical Adenomatous Hyperplasia
AIS: Adenocarcinoma in Situ
MIA: Minimally Invasive Adenocarcinoma
IAC: Invasive Adenocarcinoma
GGNs: Ground Glass Nodules
ROI: Region of Interest
VOI: Volume of Interest
mRMR: Minimum Redundancy Maximum Relevance
LASSO: Least Absolute Shrinkage and Selection Operator
RF: Random Forest
SHAP: Shapley Additive Explanations
GLSZM: Gray Level Size Zone Matrix
GLCM: Gray Level Cooccurrence Matrix
RLM: Run Length Matrix
