# Supplementary material for: A triple-classification for the evaluation of lung nodules manifesting as pure ground-glass sign: a CT-based radiomic analysis
Source: BMC Med Imaging. 2022 Jul 27;22:133. doi: 10.1186/s12880-022-00862-x (PMC9327229; doi:10.1186/s12880-022-00862-x)
Supplement: Supplementary file 1 — Additional file 1. The detail of feature selection. [file 12880_2022_862_MOESM1_ESM.docx]

| **Feature extraction** | |
| --- | --- |
| **Histogram Parameters** | |
| **Feature name** | **Mathematical definitions** |
| Energy | **The energy feature measures the uniformity of the intensity level distribution. If the value is high, then the distribution is to a small number of intensity levels.** |
| Entropy | **The entropy measures the randomness of the distribution of the coefficients values over the intensity levels.** |
| MaxIntensity | **The maximum intensity value.** |
| MinIntensity | **The minimum intensity value.** |
| MeanValue | **The mean measures the average value of the intensity values.** |
| Mean absolute deviation | **The mean of the absolute deviations of all voxel intensities around the mean intensity value.** |
| MedianIntensity | **The median intensity values.** |
| Range | **The range of intensity values.** |
| Root mean square (RMS) | **The root mean square of all voxel intensities around the mean intensity value.** |
| Standard deviation | **A measure that is used to quantify the amount of variation or dispersion of a set of data values.** |
| Uniformity | **The uniformity of all voxel intensities around the mean intensity value.** |
| Variance | **The average of the squared differences from the Mean.** |
| Volume Count | **Describe the size of the ROI.** |
| Voxel Value Sum | **Represents the Sum calculations for voxels in the ROI.** |
| RelativeDeviation | **The mean** **relative deviation of a set of quantities.** |
| Frequency Size | **The** **frequency of all voxel intensities around the mean intensity value.** |
| Quantiles | **They are cut points dividing the range of a probability distribution into contiguous intervals with equal probabilities, or dividing the observations in a sample in the same way.** |
| Percentiles | **A measure used in statistics indicating the value below which a**  **given percentage of observations in a group of observations fall.** |
| Skewness | **Represents the degree of asymmetric distribution in the image histogram, this means that in some distribution of data, the right and the left of the distribution are perfect mirror images of one another, the mean, median and mode are all measures of the center of a set of data.** |
| Kurtosis | **Kurtosis is a measure of whether the data are heavy-tailed or light-tailed relative to a normal distribution.** |
| **Form Factor Parameters** | |
| **Feature name** | **Mathematical definitions** |
| Sphericity | **A measure of how spherical the ROI.** |
| Surface area | **The surface area is calculated by triangulation.** |
| Compactness 1 | **A measure of the compactness of the ROI shape relative to the (most compact) sphere.** |
| Compactness 2 | **A measure of the compactness of the ROI shape relative to the (most compact) sphere.** |
| Maximum 3D diameter | **The maximum three-dimensional tumor diameter is measured as the largest pairwise Euclidean distance.** |
| Spherical disproportion | **The spherical proportion** |
| Surfacetovolumeratio | **The ratio between the surface area and volume of cells influences their structure and biology.** |
| Volume | **Measured by counting the number of pixels in the ROI and multiplying this value by the voxel size.** |
| VolumeCC and VolumeMM | **The maximum 3D diameter, surface area and volume provide information on the size of the lesion.** |
| **Texture Parameters** | |
| **Feature name** | **Mathematical definitions** |
| Energy | **This feature Returns the sum of squared elements.** |
| Entropy | **This is a measure of randomness.** |
| Correlation | **A measure the similarity of the grey levels in neighboring pixels** |
| Inertia | **A measure the clarity of the image and texture groove depth.** |
| Cluster Shade | **Cluster analysis or clustering is the task of grouping a set of objects in such a way that objects in the same group (cluster) are more similar (in some sense or another) to each other than to those in other groups (clusters).** |
| Cluster Prominence | **Cluster Prominence is a measure of asymmetry of a given distribution, high values of this feature indicate that the symmetry of the image is low, in medical imaging low values of cluster prominence represent a smaller peak for the image grey level value and usually the grey level difference between the forms is small.** |
| **GLCM Parameters** | |
| Energy of GLCM | **This feature Returns the sum of squared elements in the GLCM.** |
| Entropy of GLCM | **Entropy measures the loss of information or message in a transmitted signal and also measures the image information.** |
| Inertia of GLCM | **It reflects the clarity of the image and texture groove depth.** |
| Correlation | **Image-based Correlation measures the similarity of the grey levels in neighboring pixels, tells how correlated a pixel is to its neighbor over the whole image.** |
| Inverse Difference Moment | **Inverse Difference Moment (IDM) is the local homogeneity.** |
| Haralick features | |
| **Feature name** | **Mathematical definitions** |
| Haralick Correlation | **Measures the degree of similarity of the gray level of the image in the row or column direction.** |
| Angular Second Moment | **Sum of squares of each matrix element** |
| Contrast | **A measure of the contrast or the amount of local variations present in the image.** |
| Haralick Entropy | **The entropy of a gray level co-occurrence matrix (GLCM) corresponding to a given image.** |
| HaraVariance | **The variance of a gray level co-occurrence matrix (GLCM) corresponding to a given image.** |
| sumAverage | **The average sum.** |
| sumVariance | **The average variance.** |
| sumEntropy | **The average entropy.** |
| differenceVariance | **The differences of variance** |
| differenceEntropy | **The differences of Entropy** |
| inverseDifferenceMoment | **The reciprocal difference moment** |
| **RLM Parameters** | |
| **Feature name** | **Mathematical definitions** |
| **The grey level run-length matrix (RLM) 𝐏_𝐫_ (𝐢, 𝐣 \| 𝛉) is defined as the numbers of runs with**  **pixels of gray level i and run length j for a given direction θ** | |
| Short Run Emphasis | **Four directions (0°,45°,90° &135°) and two displacement vectors (1 & 4 pixel)** |
| Long Run Emphasis | **Four directions (0°,45°,90° &135°) and two displacement vectors (1 & 4** **pixel)** |
| Grey Level Non-uniformity | **Four directions (0°,45°,90° &135°) and two displacement vectors (1 & 4 pixel)** |
| Run Length Non-uniformity | **Four directions (0°,45°,90° &135°) and two displacement vectors (1 & 4 pixel)** |
| Low Grey Level Run Emphasis | **Four directions (0°,45°,90° &135°) and two displacement vectors (1 & 4 pixel)** |
| High Grey Level Run Emphasis | **Four directions (0°,45°,90° &135°) and two displacement vectors (1 & 4 pixel)** |
| Short Run Low Grey Level Emphasis | **Four directions (0°,45°,90° &135°) and two displacement vectors (1 & 4 pixel)** |
| Short Run High Grey Level Emphasis | **Four directions (0°,45°,90° &135°) and two displacement vectors (1 & 4 pixel)** |
| Long Run Low Grey Level Emphasis | **Four directions (0°,45°,90° &135°) and two displacement vectors (1 & 4 pixel)** |
| Long Run High Grey Level Emphasis | **Four directions (0°,45°,90° &135°) and two displacement vectors (1 & 4 pixel)** |
| **Discretization** | |
| Discretization methods | Fixed Bin Size (FBS) and Fixed Bin Number (FBN) |
| Discretization parameters | FBS: bin widths = 1, 5, 10, 20, 25, 50  FBN: bin numbers = 8, 16, 32, 64, 128, 256, 512, 1024 |
